# Melatonin inhibits aromatase promoter expression by regulating cyclooxygenases expression and activity in breast cancer cells

**DOI:** 10.1038/sj.bjc.6605336

**Published:** 2009-09-22

**Authors:** C Martínez-Campa, A González, M D Mediavilla, C Alonso-González, V Alvarez-García, E J Sánchez-Barceló, S Cos

**Affiliations:** 1 Department of Physiology and Pharmacology, School of Medicine, University of Cantabria, 39011 Santander, Spain

**Keywords:** melatonin, pineal, MCF-7 cells, breast cancer, aromatase

## Abstract

**Background:**

Melatonin reduces the development of breast cancer interfering with oestrogen-signalling pathways, and also inhibits aromatase activity and expression. Our objective was to study the promoters through which melatonin modifies aromatase expression, evaluate the ability of melatonin to regulate cyclooxygenases and assess whether the effects of melatonin are related to its effects on intracellular cAMP, in MCF-7 cells.

**Methods:**

Total aromatase mRNA, aromatase mRNA promoter regions and cyclooxygenases mRNA expression were determined by real-time RT–PCR. PGE_2_ and cAMP were measured by kits.

**Results:**

Melatonin downregulated the gene expression of the two major specific aromatase promoter regions, pII and pI.3, and also that of the aromatase promoter region pI.4. Melatonin 1 nM was able to counteract the stimulatory effect of tetradecanoyl phorbol acetate on PGE_2_ production and inhibit COX-2 and COX-1 mRNA expression. Melatonin 1 nM elicited a parallel time-dependent decrease in both cyclic AMP formation and aromatase mRNA expression.

**Conclusions:**

This study shows that melatonin inhibits aromatase activity and expression by regulating the gene expression of specific aromatase promoter regions. A possible mechanism for these effects would be the regulation by melatonin of intracellular cAMP levels, mediated by an inhibition of cyclooxygenase activity and expression.

Oestrogens are involved in the growth and differentiation of the normal mammary gland and have an important role in the genesis and growth of mammary tumours (Russo and Russo, 1998). Melatonin, the main hormone secreted by the pineal gland, has been shown to exert oncostatic properties on hormone-dependent mammary cancer (Cos and Sánchez-Barceló, 2000a, 2000b; Blask *et al*, 2002). Evidence from *in vivo* studies on animal models and *in vitro* studies on human breast cancer cell lines support the hypothesis that melatonin oncostatic effects on hormone-dependent mammary tumours are mainly dependent on its ability to interact with the oestrogen-signalling pathway (Hill and Blask, 1988; Cos *et al*, 2006b, 2008). At the mammary tumour cell level, melatonin interferes with the oestrogen receptor and counteracts the effects of oestrogens, thus behaving as a selective oestrogen receptor modulator (Blask and Hill, 1986; Hill and Blask, 1988). Furthermore, melatonin regulates the activity of some enzymes responsible for the local synthesis of oestrogens in human breast cancer, thus behaving as a selective oestrogen enzyme modulator (Cos *et al*, 2008; González *et al*, 2008). Using MCF-7 human breast cancer cells in culture, which express aromatase (Zhou *et al*, 1993; Sonne-Hansen and Lykkesfeldt, 2005) and the membrane-bound Gi protein-coupled receptor (MT_1_) melatonin receptor (Ram *et al*, 1998, 2002), our group has previously shown that melatonin, at physiological concentrations (1 nM), reduces aromatase activity in these cells, both under basal conditions and when aromatase activity is stimulated by cAMP or cortisol (Cos *et al*, 2005). In addition, we also demonstrated by RT–PCR that melatonin downregulates aromatase mRNA steady-state levels in MCF-7 cells (Cos *et al*, 2005). This modulator effect of melatonin on the enzyme that controls the conversion from androgenic precursors to oestrogens has also been described *in vivo* in rats bearing DMBA-induced mammary tumours (Cos *et al*, 2006a). The binding of melatonin to MT_1_ receptors has been described as the first step in the antiaromatase action of melatonin on MCF-7 cells (González *et al*, 2007).

Mammary cancer tissue contains all the enzymes responsible for the local biosynthesis of oestrogens. One of the two major pathways involved in the synthesis of oestrogens in breast cancer cells is the aromatase pathway, which transforms androgens into oestrogens (Landeghem *et al*, 1985; Yue *et al*, 1998; Simpson, 2000). It is well known that aromatase activity and expression is much higher in breast cancer tissue than in normal mammary tissue, and this is one of the reasons why oestrogen concentration in this type of tissue is highly elevated (Landeghem *et al*, 1985; Yue *et al*, 1998). This enzyme complex consists of two components: aromatase cytochrome P-450 protein and, coupled to it, a ubiquitous flavoprotein, NADPH-cytochrome P-450 reductase (Conley and Hinshelwood, 2001). The gene coding for the aromatase protein is the largest of the cytochrome P-450 family. Because its overall homology to other members of the P-450 superfamily is low, aromatase belongs to a separate gene family designated CYP19 (Santen and Harvey, 1999). Regulation of aromatase expression in human tissues is quite complex, involving alternative promoter sites that provide tissue-specific control (Bulun *et al*, 2005; Chen *et al*, 2009). In normal breast, the mammary adipose tissue maintains low levels of aromatase expression almost exclusively through promoter I.4, which is regulated by the combined action of a glucocorticoid and a member of the class I cytokine family (interleukin 6, 11). However, in mammary cancer, both in malignant epithelial cells and surrounding fibroblasts, the expression of aromatase is increased by the activation of promoters II and I.3 (Bulun *et al*, 2005; Chen *et al*, 2009). Prostaglandin E_2_ (PGE_2_) is an important regulator of aromatase gene expression through promoters II and I.3 (Bulun *et al*, 2005). As it is known (a) that melatonin, through an MT_1_, downregulates the forskolin-induced increase of cAMP in MCF-7 cells (Kiefer *et al*, 2002), and (b) that the activation of the two major promoters driving aromatase expression in breast cancer is regulated by cAMP (Bulun *et al*, 2005; Chen *et al*, 2009), the objective of this study was to expand our understanding of the antiaromatase properties of melatonin and to assess whether the promoters that drive aromatase expression are regulated by melatonin and to evaluate the ability of this indolamine to directly regulate cyclooxygenases (COX) gene expression and activity, as defined by PGE_2_ production, in MCF-7 cells. In addition, we studied whether the effects of melatonin on aromatase expression are related to the effects of this hormone on intracellular cAMP concentration.

## Materials and methods

### Cells and culture conditions

MCF-7 human breast cancer cells were purchased from the American Tissue Culture Collection (Rockville, MD, USA). They were maintained as monolayer cultures in 75 cm^2^ plastic culture flasks in Dulbecco's Modified Eagle's Medium (DMEM) (Sigma-Aldrich, Madrid, Spain) supplemented with 10% fetal bovine serum (FBS) (Gibco, France), penicillin (20 units ml^−1^) and streptomycin (20 *μ*g ml^−1^) (Sigma-Aldrich) at 37°C in a humid atmosphere containing 5% CO_2_. Cells were sub-cultured every 3–4 days by suspension in 5 mM Na_2_-EDTA in PBS (pH 7.4) at 37°C for 5 min. Before each experiment, stock sub-confluent monolayers (80%) of MCF-7 cells were incubated with 5 mM Na_2_-EDTA in PBS (pH 7.4) at 37°C for 5 min, resuspended in DMEM supplemented with 5% FBS and passed repeatedly through a 25-G needle to produce a single cell suspension. Cell number and viability were determined by staining a small volume of cell suspension with 0.4% trypan blue saline solution and examining the cells in a haemocytometer.

### Measurement of total aromatase mRNA and aromatase promoter regions pI.3, pII, and pI.4 gene expression

MCF-7 cells were grown in medium with phenol red and supplemented with 10% FBS. Before each experiment, cells were transferred to a phenol red-free DMEM medium containing 0.5% charcoal-stripped FBS and maintained for 3 days. Thereafter, analysis of total aromatase mRNA and aromatase mRNA promoter regions pI.3, pII, and pI.4 expression in MCF-7 cells was carried out by reverse transcription real-time RT–PCR, after an incubation of MCF-7 cells with either melatonin 1 nM (Sigma-Aldrich) or vehicle for 120 min.

Total cellular RNA was purified with the Nucleospin RNA II Kit (Macherey-Nagel, Düren, Germany), following the manufacturer's instructions. Integrity of RNA was assessed by electrophoresis in ethidium bromide-stained 1.2% agarose-Tris-borate EDTA gels. The absorbance ratio A_260 nm_/A_280 nm_ was greater than 1.8. For cDNA synthesis, 1 *μ*g of total RNA was denaturated at 65°C for 10 min and reverse transcribed for 50 min at 45°C with a cDNA synthesis kit (Bioline, London, UK) in a final volume of 20 *μ*l in the presence of 500 ng of oligo (dT)12–18 primer.

Primers used for the amplification of aromatase and promoter-specific transcripts of aromatase (Sigma Genosys Ltd., Cambridge, UK), using the housekeeping gene S14, are listed in Table 1. The primers were designed so that the coding sequence between the two PCR primer sites is interrupted by at least one intron in the gene.

Real-time PCRs were performed in an MX3000 (Stratagene, La Jolla, CA, USA) using Brilliant SYBR Green PCR Master Mix (Stratagene), following the manufacturer's instructions. Amplifications were performed for 40 cycles using the following temperature profile: 58°C, 60 s (annealing); 72°C, 30 s (extension) and 95°C, 30 s (denaturation).

In some experiments, to confirm the relationship between PGE_2_ and aromatase expression, cells were incubated for 120 min with PGE_2_ 10 *μ*M, melatonin 1 nM and luzindole 10 *μ*M (all from Sigma-Aldrich) before measuring total aromatase mRNA expression. In addition, the effects of cAMP-modulating agents (8-Br-cAMP and RP-cAMP; Sigma-Aldrich) on aromatase mRNA steady-state levels were determined in cells treated with these agents (1 *μ*M) in the presence or absence of melatonin 1 nM.

### Assay of COX enzymatic activity and PGE_2_ measurement

To study COX enzymatic activity, as defined by PGE_2_ synthesis in culture media, the experiments were performed in 12-well plates. MCF-7 cells (150 000 cells) were added to each well and plates were maintained overnight in DMEM supplemented with 10% FBS, penicillin (20 units ml^−1^) and streptomycin (20 *μ*g ml^−1^), at 37°C, to allow the cells to adhere to the plates. Cells were then serum starved in defined media for 24 h. Thereafter, the media were changed for fresh ones supplemented with 100 *μ*M sodium arachidonate (Sigma-Aldrich) and containing tetradecanoyl phorbol acetate (TPA) 10 nM (Sigma-Aldrich), melatonin 1 nM or the diluent of these drugs (ethanol, at a final concentration lower than 0.0001% per plate). After 5 h incubation at 37°C, the media were collected, centrifuged to obtain a cell-free fraction and frozen at −70°C until assays were performed. PGE_2_ concentration in the supernatant of culture media was determined by ELISA (Cayman Chemical Company, Ann Arbor, MI, USA), according to the protocol provided by the manufacturer.

### Measurement of COX-1 and COX-2 mRNA expression

Analysis of COX-1 and COX-2 mRNA expression in MCF-7 cells was carried out by reverse transcription real-time RT–PCR after incubating MCF-7 cells with either melatonin 1 nM or vehicle for 120 min. Total cellular RNA was purified with the Nucleospin RNA II Kit (Macherey-Nagel, Düren, Germany) following the manufacturer's instructions. Integrity of RNA was assessed by electrophoresis in ethidium bromide-stained 1.2% agarose-Tris-borate EDTA gels. The absorbance ratio A_260 nm_/A_280 nm_ was greater than 1.8. For cDNA synthesis, 1 *μ*g of total RNA was denaturated at 65°C for 10 min and was reverse transcribed for 50 min at 45°C using a cDNA Synthesis kit (Bioline, London, UK) in a final volume of 20 *μ*l in the presence of 500 ng of oligo (dT)12–18 primer.

PCR was performed using a set of human COX-1- and COX-2-specific primers (Sigma Genosys Ltd.) listed in Table 1. The coding sequence between the two PCR primer sites is interrupted by at least one intron in the gene. As a control quantification, S14 mRNA was also carried out by RT–PCR using a set of specific primers (Table 1).

Real-time PCRs were performed in an MX3000 (Stratagene) using Brilliant SYBR Green PCR Master Mix (Stratagene) following the manufacturer's instructions. Amplifications were performed for 40 cycles using the following temperature profile: 58°C, 60 s (annealing); 72°C, 30 s (extension); and 95°C, 30 s (denaturation).

### Measurement of cAMP levels

MCF-7 cells were grown in a medium supplemented with 10% FBS for 3 days in the presence of melatonin 1 nM or vehicle. Thereafter, cAMP was analysed using a commercial kit, the Cyclic AMP [^3^H] Assay System (Amersham Biosciences, Barcelona, Spain).

### Statistical analysis

Data are expressed as mean±s.e.m. Differences in aromatase mRNA promoter regions pI.3, pII, and pI.4, and COX-1 and COX-2 mRNA expression, were analysed by unpaired Student's *t*-test. Comparisons of PGE_2_ levels and total aromatase mRNA expression were carried out using one-way analysis of variance, followed by the Student–Newman–Keuls test. Results were considered as statistically significant at *P*<0.05.

## Results

### Effects of melatonin on aromatase promoter regions pI.3, pII, and pI.4 expression

**Figure 1:**
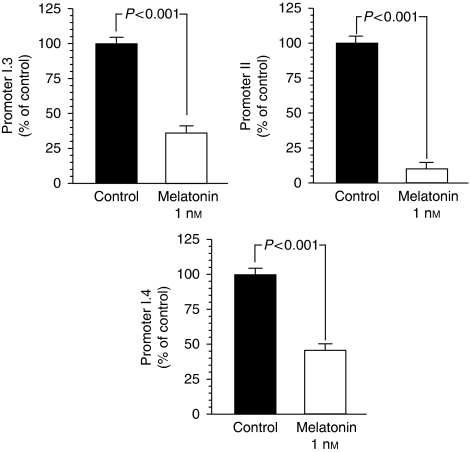
RT–PCR analysis of the gene expression of aromatase promoter regions pI.3, pII, and pI.4 in MCF-7 cells after 120 min incubation with melatonin (1 nM) or ethanol (0.001%)(control). Data are expressed as the percentage of the control group.

To determine the promoters involved in the melatonin control of aromatase expression, we used competitive RT–PCR to amplify each of the promoter-specific transcripts from RNA extracted from MCF-7 cells. As shown in Figure 1, melatonin 1 nM exerted a significant (*P*<0.001) and potent inhibition in aromatase expression that is specific to the aromatase promoters II, pI.3, and pI.4.

### Effect of melatonin on COX enzymatic activity and production of PGE_2_

**Figure 2:**
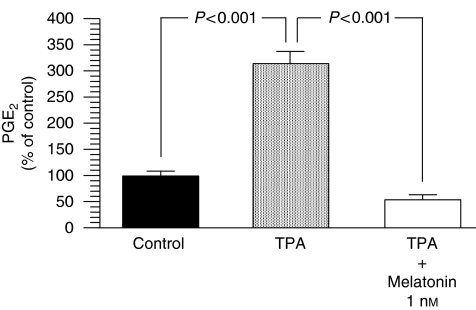
Effects of melatonin (1 nM) on COX enzymatic activity as defined by PGE_2_ production after tetradecanoyl phorbol acetate (TPA) stimulation. Data are expressed as a percentage of the control group (mean±s.e.m.).

PGE_2_ production by MCF-7 cells was measured by ELISA to determine whether cyclooxygenase enzyme activity was modified after treating cells with physiological concentrations of melatonin (1 nM). The levels of COX activity in MCF-7 cells were low but detectable. Tetradecanoyl phorbol acetate (TPA) is an exogenous natural product showing tumour-promoting activity, which, through the stimulation of protein kinase C activity, induces COX enzymatic activity and increases the synthesis of PGE_2_. Melatonin 1 nM was able to significantly counteract (*P*<0.001) the stimulatory effect of TPA on PGE_2_ production by decreasing the PGE_2_ concentration to levels similar to those found in culture media of control cells (Figure 2).

### Effects of melatonin on COX-1 and COX-2 mRNA expression

**Figure 3:**
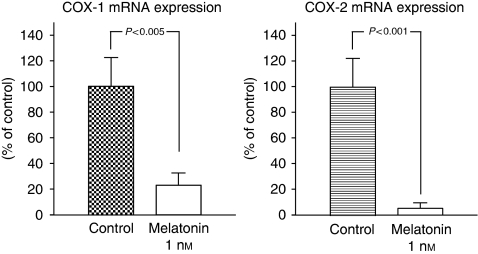
Effects of melatonin (1 nM) or the diluent (ethanol at 0.0001% final concentration) on COX-1 and COX-2 mRNA expression. Data are expressed as a percentage of the control group (mean±s.e.m.).

The levels of COX-1 and COX-2 expression in MCF-7 cells were studied by real-time RT–PCR. Melatonin at physiological (1 nM) doses significantly (*P*<0.001) inhibited the expression of COX-2 by as much as 10 times (Figure 3). The COX-1 mRNA expression was also significantly (*P*<0.005) reduced by melatonin by as much as four times (Figure 3).

### Effects of melatonin on aromatase mRNA expression induced by PGE_2_

**Figure 4:**
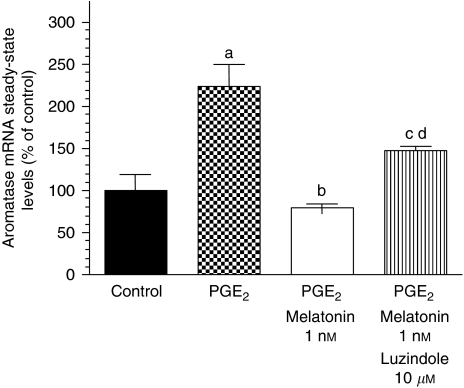
Effects of melatonin (1 nM) on aromatase mRNA steady-state levels induced by prostaglandin E_2_ (PGE_2_). Data are expressed as a percentage of the control group (mean±s.e.m.). a, *P*<0.001 *vs* that of control; b, *P*<0.001 *vs* that of PGE_2_; c, *P*<0.05 *vs* that of PGE_2_; d, *P*<0.05 *vs* that of PGE_2_+melatonin 1 nM.

Figure 4 shows that 1 nM melatonin also significantly (*P*<0.001) decreased the aromatase mRNA steady-state levels induced by PGE_2_, a well-known inducer of aromatase expression. The addition of luzindole 10 *μ*M, a melatonin receptor antagonist, prevented the inhibitory effect of melatonin on aromatase mRNA steady-state levels, thus suggesting a potential involvement of melatonin receptors in these effects.

### Effects of melatonin on aromatase mRNA expression and intracellular c-AMP levels

**Figure 5:**
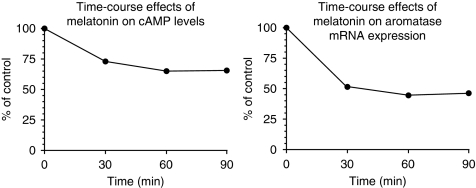
Time course of inhibition of cAMP levels and aromatase mRNA expression by melatonin (1 nM). Data are expressed as a percentage of the control group.

Figure 5 shows the correlation between time-course changes in cAMP and aromatase expression after melatonin treatment. As observed, melatonin 1 nM elicited a parallel time-dependent decrease in both cyclic AMP formation and aromatase mRNA expression.

**Figure 6:**
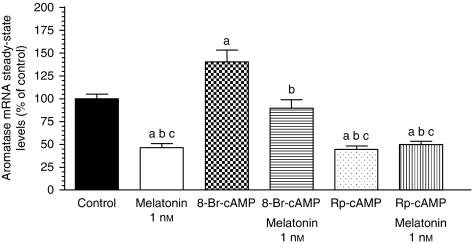
Effects of melatonin on both aromatase mRNA steady-state and intracellular cAMP levels. Data are expressed as a percentage of the control group (mean±s.e.m.). a, *P*<0.05 *vs* that of control; b, *P*<0.05 *vs* that of 8-Br-cAMP; c, *P*<0.05 *vs* that of 8-Br-cAMP+melatonin 1 nM.

To determine whether the effects of melatonin on aromatase mRNA steady-state levels were dependent (not only coincident) on the effects of the hormone on intracellular cAMP, we studied aromatase mRNA steady-state levels from cells treated with agents that should mimic (Rp-cAMP) or block (8-Br-cAMP) these effects of melatonin. Figure 6 shows that melatonin significantly (*P*<0.05) inhibited aromatase mRNA steady-state levels similar to Rp-cAMP, an inhibitor of the activation by cAMP. In contrast, 8-Br-cAMP, a membrane-permeable cAMP analogue significantly (*P*<0.05) increased aromatase mRNA steady-state levels. When cells were also grown in the presence of 1 nM melatonin, the aromatase mRNA expression was significantly (*P*<0.05) lower than in those treated only with 8-Br-cAMP.

## Discussion

The relevance of oestrogens in the genesis and progression of breast cancer is supported by important experimental and epidemiological evidence (Russo and Russo, 1998). Ovaries are the main sites of oestrogen synthesis in premenopausal non-pregnant women. However, after menopause, local synthesis of oestrogens in some tissues, including mammary tissue, acquires a special importance in mammary carcinogenesis (Ackerman *et al*, 1981; Pasqualini, 2004; Pasqualini and Chetrite, 2005; Suzuki *et al*, 2005). The local synthesis of oestrogens depends on the activity of different enzyme families (aromatase, sulfatases, etc) to transform androgens into oestrogens, as well as compounds of weak oestrogenic activity into more active forms. All these enzymes contribute to the regulation of the oestrogen availability in breast tumours and in other kinds of tumours, such as in human endometrial carcinoma (Pasqualini, 2004; Pasqualini and Chetrite, 2005; Suzuki *et al*, 2005). This is the reason why a new therapeutic strategy, based on the inhibition of oestrogen synthesis (drugs collectively known as selective oestrogen enzyme modulators), is currently being developed for breast cancer treatment (Wong and Ellis, 2004).

At present, the validity of melatonin as an oncostatic agent, especially in hormone-dependent mammary tumours, is well established (Blask *et al*, 2002; Cos *et al*, 2006b, 2008). Melatonin exerts oncostatic effects on breast cancer by interacting with oestradiol at the oestrogen receptor level (Cos and Sánchez-Barceló, 2000a, 2000b; Del Rio *et al*, 2004; Cos *et al*, 2006b) and by regulating the activity and expression of some enzymes responsible for the local synthesis of oestrogens (Cos *et al*, 2005, 2006b, 2008). As we previously showed that melatonin inhibits aromatase activity and expression, both *in vitro* in culture of human breast cancer cells (Cos *et al*, 2005) and *in vivo* in rats bearing DMBA-induced mammary tumours (Cos *et al*, 2006a), we wanted to expand our understanding of the antiaromatase properties of melatonin; therefore, our objective now was to assess which promoters of the aromatase gene are modified by melatonin. Furthermore, as there is an association between high cyclooxygenase mRNA expression and upregulation of the gene expression of the two major specific aromatase promoter regions, pII and pI.3, in breast tumours (Díaz-Cruz *et al*, 2005; Prosperi and Robertson, 2006), we were also interested in evaluating the ability of melatonin to directly regulate cyclooxygenase gene expression and activity as a possible mechanism for the modulation of the aromatase enzyme.

In disease-free breast tissue, aromatase is primarily expressed in adipose stromal fibroblasts by the relatively weak promoter I.4. However, in breast cancer tissue, aromatase promoters I.3 and II are activated, leading to a marked increase in aromatase expression in malignant epithelial cells and adipose fibroblasts. Both promoters are considered to be the major promoters driving aromatase expression in breast cancer and surrounding adipose tissue, accounting for 80–90% of total aromatase expression (Bulun *et al*, 2005; Chen *et al*, 2009). This study shows that melatonin-dependent regulation of aromatase gene expression occurs mainly through a downregulation of gene expression of the specific aromatase promoter regions pII and pI.3 in breast cancer cells. Aromatase pII was the most abundantly expressed compared with either pI.3 or pI.4, and melatonin inhibitory action was more potent on the aromatase promoter region pII. Furthermore, melatonin also decreased the aromatase promoter region's pI.4 expression in MCF-7 cells. The switch in the regulatory mechanism of aromatase expression from normal breast tissue to cancerous tissue has been extensively investigated, and it has been described that promoters II and I.3 are both regulated by cAMP and factors that regulate cAMP pathways (Bulun *et al*, 2005; Chen *et al*, 2009). One factor that has been described to regulate the activity of the aromatase enzyme through the activation of promoter II of the aromatase gene is the bioactive lipid prostaglandin E_2_ (Díaz-Cruz *et al*, 2005; Prosperi and Robertson, 2006). A model to explain the interrelationship between aromatase and COX enzymes has been proposed. High levels of expression of COX enzymes and increased COX activity result in higher levels of PGE_2_. Elevated PGE_2_ levels increase intracellular cAMP and result in an increased aromatase expression by the activation of promoters I.3 and II. The levels of PGE_2_ in the culture medium of MCF-7 cells were low but detectable, and TPA, which is an inducer of COX enzymatic activity through the stimulation of protein kinase C activity, increased the synthesis of PGE_2_. Melatonin 1 nM was able to counteract significantly the stimulatory effect of TPA on PGE_2_ production, by decreasing PGE_2_ concentration similar to levels found in culture media of control cells. The formation of prostaglandins occurs through the activity of cyclooxygenases, rate-limiting enzymes that catalyse the conversion of arachidonic acid to prostaglandins. There are two isoforms, COX-1 and COX-2. COX-1 is constitutively expressed in most tissues and considered to generate prostaglandins for normal functions (Liu and Rose, 1996). Although COX-1 is present at a constant level in most cells and tissues, some studies have shown that COX-1 activity and expression is also elevated in human breast cancer tumours, prostatic tumours and in squamous cell carcinomas of the oropharynx, hypopharynx and larynx (Liu and Rose, 1996; Hwang *et al*, 1998; Erovic *et al*, 2008). COX-2 is present in breast cancer tissue but not in normal breast tissue, and can undergo rapid induction in response to a variety of stimuli, including mitogens, hormones, cytokines and growth factors (Díaz-Cruz *et al*, 2005). Prostaglandins produced by COX-2, predominantly prostaglandin E_2_, induce inflammation and are potent mediators of a number of signal transduction pathways that are implicated in cancer development. Melatonin at physiological (1 nM) doses inhibited the expression of COX-2 in MCF-7 cells by as much as 10 times (10% of controls). The expression of COX-1 mRNA expression was also significantly reduced by melatonin by as much as four times (20% of controls). This study shows that melatonin decreases COX enzymatic activity and this inhibitory action begins at the transcriptional level by inhibiting the expression of COX-1 and, above all, COX-2. Overexpression of COX-2 has been shown to have a key effect in inflammatory disorders, tissue injury and tumourigenesis, and several endogenous molecules and natural products, such as melatonin, salicylates or polyphenols, have been reported to inhibit COX-2 expression by targeting the transcriptional activation induced by pro-inflammatory mediators (Wu, 2005). In particular, melatonin has been shown in a carrageenan-induced inflammation rat model to protect inflammatory tissue injury, and its protective effect was reported to be correlated with the suppression of COX-2 expression and COX-2-derived prostaglandins in inflamed tissues (Wu, 2005). Recently, by using computer graphics applications, it has been described that melatonin has an excellent steric and electronic effect that is complementary with COX and, therefore, it seems possible that melatonin might bind to the active site of COX-1 and COX-2, modulating the activity of this enzyme (De la Rocha *et al*, 2007). Anti-inflammatory actions of melatonin, in which this indolamine specifically prevents the activation of the COX-2 enzyme, have also been described *in vitro* in cultures of C6 glioma cells (Esposito *et al*, 2008) and in macrophages (Mayo *et al*, 2005). Melatonin at physiological (1 nM) doses reduced aromatase mRNA steady-state levels both under basal conditions and when aromatase mRNA expression was stimulated by adding PGE_2_. The inhibitory effect of melatonin was reverted by luzindole and cells had levels of aromatase mRNA expression similar to control cells, indicating that melatonin acts through known melatonin receptor-mediated mechanisms.

Melatonin, through a membrane-bound Gi protein-coupled receptor (MT_1_), downregulates cAMP in different cell types (Godson and Reppert, 1997; Kiefer *et al*, 2002). In MCF-7 cells, it has been shown that melatonin at a nanomolar concentration reduces the forskolin-induced increase of cAMP (Kiefer *et al*, 2002) and, in murine mammary tissue, melatonin decreases cAMP and increases cGMP in both a dose- and time-dependent manner (Cardinali *et al*, 1992). Furthermore, the activation of the two major promoters driving aromatase expression in breast cancer is regulated by cAMP and by factors that regulate cAMP levels (Bulun *et al*, 2005; Chen *et al*, 2009). When we compared the time course of the action of melatonin on cyclic AMP formation with the time course of melatonin on aromatase mRNA expression, we found that melatonin 1 nM elicited a time-dependent decrease in both cyclic AMP formation and aromatase mRNA expression. Furthermore, melatonin inhibition of aromatase mRNA expression is similar to that elicited by Rp-cAMP, an inhibitor of activation by cAMP, whereas melatonin counteracted the stimulatory effect of an analogue of cAMP (8-Br-cAMP).

Although promoter I.4 is less important in breast cancer, MCF-7 cells express this promoter, and melatonin 1 nM was able to decrease its expression. Promoter I.4 is regulated by a combined action of a glucocorticoid and a member of the class I cytokine family (interleukin 6, 11) (Bulun *et al*, 2005; Chen *et al*, 2009). Because it is known that melatonin represses dexamethasone-induced activation of the glucocorticoid receptor in MCF-7 cells (Kiefer *et al*, 2005), this melatonin regulation of glucocorticoid receptor transcriptional activity could be a link between melatonin and inactivation of aromatase promoter region pI.4 expression.

Taken together, all these results demonstrate that one of the mechanisms through which melatonin can modulate aromatase enzyme in breast tumour cells is through its downregulatory action on the expression of COX enzymes, which decrease the levels of PGE_2_. Lower levels of PGE_2_ decrease intracellular levels of cAMP, which in turn decrease the activation of promoters I.3 and II and result in decreased aromatase expression. Lower levels of aromatase lead to lower levels of oestrogens, resulting in decreased growth and development of breast tumour.

In conclusion, this study shows that melatonin inhibits aromatase activity and expression by regulating the gene expression of specific aromatase promoter regions, and at least one mechanism through which this could occur is the melatonin regulation of intracellular cAMP levels, mediated by a modulation of cyclooxygenase activity and expression induced by melatonin.

## Figures and Tables

**Table 1:** Primers used for RT–PCR detection of aromatase, aromatase promoter regions pI.3, pI.4, and pII, cyclooxygenases 1 and 2 and S14 (control)

**mRNA**	**Sequence**
Aromatase (F)	5′–TATTGGAAATGCTGATTGCGG
Aromatase (R)	5′-TTGGGCTTGGGGAAATACTCG
pI.3 (F)	5′-GGGCTTCCTTGTTTTGACTGTAA
pI.3 (R)	5′-AGAGGGGGCAATTTAGAGTCTGTT
pII (F)	5′-CTCTGAAGCAACAGGAGCTATAGA
pII (R)	5′-CAGGCACGATGCTGGTGATG
pI.4 (F)	5′-AACGTGACCAACTGGAGCCTG
pI.4 (R)	5′-CATCACCAGCATCGTGCCTG
COX-1 (F)	5′-ACCCGCACGGGCTATTCCGGC
COX-1 (R)	5′-AGGCGCATGAGCATCTCTCGG
COX-2 (F)	5′-ATGTATGAGTGTGGGATTTGA
COX-2 (R)	5′-TCCAAAATCCCTTGAAGTGGG
S14 (F)	5′-TCACCGCCCTACACATCAAAC
S14 (R)	5′-TCCTGCGAGTGCTGTCAGAG

(F) sense forward primer; (R) antisense (reverse) primer.
